# Gene expression profile predictive of response to chemotherapy in metastatic colorectal cancer

**DOI:** 10.18632/oncotarget.3152

**Published:** 2015-01-30

**Authors:** Purificacion Estevez-Garcia, Fernando Rivera, Sonia Molina-Pinelo, Marta Benavent, Javier Gómez, Maria Luisa Limón, Maria Dolores Pastor, Julia Martinez-Perez, Luis Paz-Ares, Amancio Carnero, Rocio Garcia-Carbonero

**Affiliations:** ^1^ Laboratorio de Oncología Molecular y Nuevas Terapias, Instituto de Biomedicina de Sevilla (IBIS) (HUVR, CSIC, Universidad de Sevilla), Sevilla, Spain; ^2^ Medical Oncology Department, Hospital Universitario Virgen del Rocio, Sevilla, Spain; ^3^ Medical Oncology Department, Hospital Universitario Marques de Valdecilla, Santander, Spain; ^4^ Pathology Department, Hospital Universitario Marques de Valdecilla, Santander, Spain; ^5^ Laboratorio de Biología Molecular del Cáncer, Instituto de Biomedicina de Sevilla (IBIS) (HUVR, CSIC, Universidad de Sevilla), Sevilla, Spain

**Keywords:** Colorectal cancer, chemotherapy, gene expression, predictive, microarray

## Abstract

Fluoropyrimidine-based chemotherapy (CT) has been the mainstay of care of metastatic colorectal cancer (mCRC) for years. Response rates are only observed, however, in about half of treated patients, and there are no reliable tools to prospectively identify patients more likely to benefit from therapy. The purpose of our study was to identify a gene expression profile predictive of CT response in mCRC. Whole genome expression analyses (Affymetrix GeneChip^®^ HG-U133 Plus 2.0) were performed in fresh frozen tumor samples of 37 mCRC patients (training cohort). Differential gene expression profiles among the two study conditions (responders versus non-responders) were assessed using supervised class prediction algorithms. A set of 161 differentially expressed genes in responders (23 patients; 62%) versus non-responders (14 patients; 38%) was selected for further assessment and validation by RT-qPCR (TaqMan^®^Low Density Arrays (TLDA) 7900 HT Micro Fluidic Cards) in an independent multi-institutional cohort (53 mCRC patients). Seven of these genes were confirmed as significant predictors of response. Patients with a favorable predictive signature had significantly greater response rate (58% vs 13%, *p* = 0.024), progression-free survival (61% vs 13% at 1 year, HR = 0.32, *p* = 0.009) and overall survival (32 vs 16 months, HR = 0.21, *p* = 0.003) than patients with an unfavorable gene signature. This is the first study to validate a gene-expression profile predictive of response to CT in mCRC patients. Larger and prospective confirmatory studies are required, however, in order to successfully provide oncologists with adequate tools to optimize treatment selection in routine clinical practice.

## INTRODUCTION

Colorectal cancer (CRC) is the third most common tumor in the world and is responsible for 8% of cancer related deaths [1]. Although prognosis has greatly improved over the past decades due to significant surgical and medical advances, once the tumor has progressed beyond surgical resectability the disease is essentially incurable. Several combination regimens including fluoropyrimidines and oxaliplatin and/or irinotecan, with or without monoclonal antibodies targeting VEGF or EGFR, remain the mainstay of care in metastatic CRC (mCRC). Response rates, however, are observed in only 40–60% of the patients and median survival does not generally exceed 24 months [2, 3, 4]. As treatment options expand, the development of reliable tools to discriminate patients likely to benefit from specific therapies remains a major clinical challenge. Indeed, with the exception of RAS mutations as predictors of resistance to EGFR-targeted therapy, no validated biomarker to date has been able to assist clinicians in the selection of the most appropriate treatment regimen for a specific patient [5, 6].

Gene expression profiling has demonstrated great potential in cancer research, improving diagnostic, prognostic and predictive precision in several tumor types [7], including CRC [8]. Over the last two decades, different studies have proved that gene expression profiles are able to discriminate normal colonic tissue from benign adenomas and adenocarcinomas in different stages of tumor progression [9], or to stratify the risk of developing CRC of normal colonic tissue [10]. More recently, different gene signatures developed in early-stage CRC have shown to predict the risk of relapse in these patients [11–14], and some of them have demonstrated improved prognosis accuracy over conventional clinical and pathological features [15]. By contrast, however, the value of this technology to predict response to therapy has not been deeply investigated. Although a number of studies have successfully identified gene signatures able to predict sensitivity to different agents in CRC cell lines [16, 17], studies assessing the potential predictive role of gene profiling in patients with advanced disease are scarce [18–20]. Therefore, larger and validation studies are needed to generate reliable data capable to make the desirable transition to the clinic.

Our aim in this project was to generate a gene profile predictor of response to chemotherapy in patients with mCRC treated with fluoropyrimidine-based regimens. Whole genome expression analyses were performed in tumor samples of mCRC patients and differentially expressed genes were then validated by RT-qPCR in an independent cohort of mCRC patients.

## RESULTS

### Gene expression profile development (training cohort)

**Figure 1:**
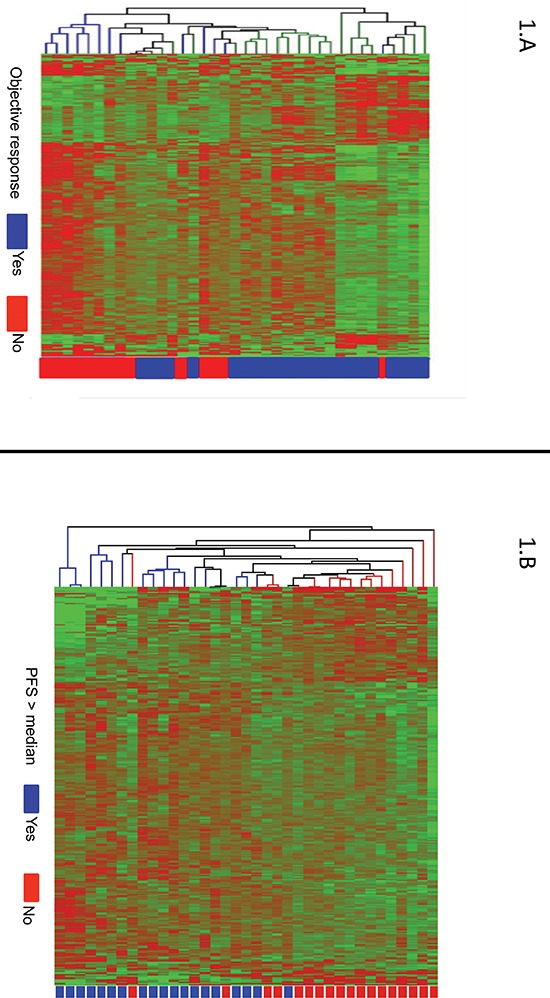
(A) Supervised hierarchical cluster analysis showing differentially expressed genes in patients achieving an objective response to chemotherapy (Yes: CR or PR; blue) versus patients non-responding to chemotherapy (No: SD or PD; red) Genes in red indicate overexpression; those in green indicate underexpression. **(B)** Supervised hierarchical cluster analysis showing differentially expressed genes in patients achieving a progression free survival (PFS) higher than the median of PFS (Yes; blue) versus patients with a PFS lower than the median (No; red). Genes in red indicate overexpression; those in green indicate underexpression.

Whole human genome expression profiles were assessed in tumor samples of patients in the training cohort (*N* = 37) using Affymetrix U133 Plus 2.0 chips. All of the samples provided adequate RNA for microarray analysis. Supervised analysis identified 595 differentially expressed genes (*p* < 0.05) in responders (23 patients; 62%) versus non-responders (14 patients; 38%) (Figure 1A). In addition, when supervised analyses were performed with PFS as a surrogate marker for response (Figure 1B), 318 genes were identified to be differentially expressed in patients with long (> median PFS) versus short (≤ median PFS) PFS values. Among the top 250 genes with greater statistical significance (lower *P* values) in cluster analyses using objective response to chemotherapy as the primary outcome measure, a set of 161 genes were selected for further validation by RT-qPCR based on the greater magnitude of their fold-change values, the degree of concordance for both outcome measures (objective response and PFS), and their biological relevance in CRC. A detailed list of the 161 selected genes is depicted in supplementary Table S3.

### Validation of the gene signature predictive of response to chemotherapy in mCRC patients

Selected genes were assessed by Taqman-based RT-qPCR in tumor samples of the validation multi-institutional cohort of mCRC patients. As depicted in Table 1, 7 of these genes were validated to be differentially expressed among patients that achieved an objective response to chemotherapy (R: CR + PR) versus those that did not (NR: SD + PD) using the Statminer^®^ software v.4.2 (adjusted *p* values < 0.05): *DCK, DNAJC3, NAV1, NIPBL, PALM2, VSNL1* and *WSHC1L1*.

**Table 1:** Differentially expressed genes by chemotherapy response in patients with metastatic colorectal carcinoma according to real-time PCR analysis

Gene	Gen ID	R vs NR(−ΔΔCt)	Adjusted *P*-values^*^	Fold-change
***DCK***	1633	1.300	**0.035**	**2.46**
***DNAJC3***	5611	1.621	**0.008**	**3.08**
***NAV1***	89796	1.231	**0.035**	**2.35**
***NIPBL***	25836	1.310	**0.035**	**2.48**
***PALM2***	445815	1.237	**0.040**	**2.36**
***VSNL1***	7447	1.552	**0.009**	**2.93**
***WHSC1L1***	54904	1.249	**0.035**	**2.38**

Patients treated with chemotherapy were stratified into one of two groups: 1) chemotherapy responders (R), including patients with complete response or partial response or 2) chemotherapy non-responders (NR), including patients with stable disease or progressive disease, according to RECIST 1.1 criteria.

* The resulting *p*-values were adjusted for multiple testing by Benjamini-Hochberg adjustment.

Gene ID: Genbank accession number.

### Risk score according to the 7-gene signature

**Figure 2:**
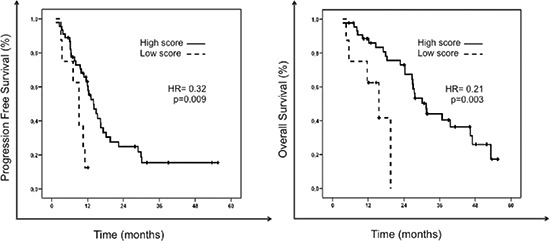
Progression free survival (PFS) and overall survival (OS) of patients according to the 7-gene score predictive of response to chemotherapy The solid black line represents patients with high score or favorable predictive signature: those with favorable gene expression levels (above the median) in ≥ 4 genes of the signature. The dashed black-line represents patients with low score or unfavorable predictive signature: those with favorable gene expression levels in ≤ 3 genes of the signature.

Following independent validation of the 7 genes, a risk score was developed to classify each patient as high score or favorable predictive signature, if they had favorable gene expression levels in at least 4 of the 7 genes in the signature, or low score or unfavorable predictive signature, if they had favorable gene expression levels in ≤ 3 of the 7 genes in the signature. Patients with a favorable predictive signature had a significantly greater response rate (58% vs 13%, *p* = 0.024) and PFS (61% vs 13% at 1 year, HR = 0.32, *p* = 0.009) than patients with an unfavorable predictive signature. Overall survival was also significantly longer for patients with high versus low score signatures (32 vs 16 months, HR = 0.21, *p* = 0.003). Figure 2 illustrates PFS and OS of patients according to the 7-gene score.

### Biological and molecular function of validated predictive genes

According to the GeneOntology [21] classification, these genes are involved in the following biological functions or molecular pathways: control of cell shape or adhesion (PALM2), transcription regulation (WSHC1L1), stem cell maintenance (NIPBL), chaperone binding (DNAJC3), nucleotide and folic acid metabolism (DCK), microtubule bindle formation (NAV1), and ion transport and binding (VSNL1). Detailed description of genes and the functional classes to which they belong is outlined in Table 2.

**Table 2:** Gene profile that predicts response to chemotherapy in mCRC patients

Probe Set Identification	Gene Symbol	Official Name	Gene ID	GO Biological Processes (BP) and Molecular Function (MF) Description
203302_at	*DCK*	deoxycytidine kinase	1633	BP: Nucleotide metabolism MF: Deoxycytidine kinase activity, ATP binding
208499_s_at	*DNAJC3*	DnaJ (Hsp40) homolog, subfamily C, member 3	5611	BP: Defense response to virus, catabolic process MF: chaperone binding, misfolded protein binding, protein kinase inhibitor
224771_at	*NAV1*	neuron navigator 1	89796	BP: neuron migration, microtubule bindle formation MF: nucleotide binding
242352_at	*NIPBL*	Nipped-B homolog	25836	BP: embryonal development, stem cell maintenance, response to DNA damage stimulus, negative regulation of transcription DNA dependent, positive regulation of hystone deacetylation MF: chromatin binding, hystone deacetylase binding, mediator complex binding, protein binding (C-terminus and N-terminus)
202760_s_at	*PALM2*	PALM2-AKAP2 readthrough	445815	BP: regulation of cell shape
203798_s_at	*VSNL1*	visinin-like 1	7447	MF: calcium ion binding
222544_s_at	*WHSC1L1*	Wolf-Hirschhorn syndrome candidate 1-like 1	54904	BP: cell differentiation and growth, histone lysine methylation, regulation of transcription DNA dependent MF: histone-lysine N-methyltransferase activity, zinc ion binding

## DISCUSSION

In this study we developed a gene expression profile able to predict treatment response in mCRC patients treated with fluoropyrimidine-based standard chemotherapy regimens. Seven genes were independently validated to be significantly overexpressed in patients achieving an objective response to chemotherapy. A risk score was developed with these 7 genes that was able to prospectively discriminate those patients most likely to benefit from therapy. Indeed, patients with a favorable predictive signature (favorable gene expression levels in at least 4 of the 7 genes in the signature) had a significantly greater response rate (58% vs 13%, *p* = 0.024), PFS (61% vs 13% at 1 year, HR = 0.32, *p* = 0.009) and OS (32 vs 16 months, HR = 0.21, *p* = 0.003) than patients with an unfavorable predictive signature. This is to our knowledge the first study to validate a gene-expression profile predictive of response to chemotherapy in advanced colorectal patients.

Many attempts have been made over the past decades to identify molecular markers predictive of response to chemotherapy in the context of CRC. Altered gene or protein expression of a number of genes have been associated with drug cytotoxicity, including thymidylate synthase, dihydropyrimidine dehydrogenase or thymidine phosphorylase for 5FU, topoisomerase I for irinotecan, or excision repair cross-complementing 1 (ERCC1) for oxaliplatin. However, none of these putative markers have been implemented in clinical practice due to their poor prediction accuracy and also to the lack of reproducibility across different studies and patient populations. These discrepancies are not unexpected, as sensitivity to treatment is a complex issue dependent on many individual and tumor factors, that ultimately determine, among other critical issues, drug disposition and pharmacodynamic effects on normal and malignant cells, as well as cell response to drug damage. The common use of multiple-drug regimens further complicates this scenario. In this context, multiple-gene signatures are likely to improve prediction accuracy over single marker genes [8, 15, 16]. However, and in spite of the undeniable success of several microarray-based prognostic gene signatures, currently being validated in prospective clinical trials [22–26] (i.e. Mammaprint and Oncotype DX in breast cancer, or Coloprint in CRC), predictive genomics remain a challenge.

Our study validated several predictive genes implicated in key cellular pathways and also some genes with molecular functions potentially related to chemotherapy response. Deoxycytidine kinase (DCK) is required for the phosphorylation of several deoxyribonucleosides and their nucleoside analogs, which are widely used as antiviral and anticancer agents (i.e. cytarabine, gemcitabine). Increased DCK activity is associated with increased activation of these compounds to cytotoxic nucleoside triphosphate derivatives, and DCK deficiency correlated with resistance to these agents in a panel of hematologic and solid cell lines *in vitro*, and also in some human tumors [27]. VSNL1 is a member of the visinin/recoverin subfamily of neuronal calcium sensor proteins, mainly expressed in the central nervous system, that modulate intracellular signaling pathways by regulating the activity of adenyl cyclase. Upregulation of VSNL1 potentiated the anoikis-resistant ability of neuroblastoma tumor cells and enhanced neuroblastoma cell invasiveness and metastasis [28]. Some authors have also suggested VSNL1 may play an important role in the invasive phenotype of CRC, and may also influence sensitivity to cytotoxic agents active in this disease, such as campthotecins [29]. Indeed, VSNL1 overexpression was associated with a higher risk of lymphatic invasion and a poorer prognosis in a series of patients with early stage CRC [30], and downregulation of this gene was observed in camptothecin-resistant gastric cancer cell lines [29]. Finally, PALM2 has been reported to be upregulated in responder CRC tumors to MS-275, a selective histone deacetylase inhibitor that disturbs cell adhesion, response to extracellular stimuli and transcription cellular processes [31]. However, the function of the putative protein product of PALM2-AKAP2, a naturally occurring cotranscribed mRNA, remains to be elucidated.

While gene expression profiling has been widely applied to CRC for diagnosis, classification and prognosis, studies evaluating its potential role to predict response to medical therapy are still scarce. Indeed, most available data to date derive from preclinical studies. Mariadason and colleagues demonstrated the ability of gene profiling to predict 5FU- and irinotecan-induced apoptosis in a panel of 30 CRC cell lines [16], and, importantly, they showed how this approach predicted response more accurately than 4 previously established determinants of 5FU response: thymidylate synthase, thymidine phosphorylase, mismatch repair status and p53 mutation [16]. Other investigators have also reported gene signatures predictive of 5FU or oxaliplatin sensitivity *in vitro* [17]. However, very few studies have assessed the predictive role of gene profiling in the clinical setting (i.e. patients with advanced CRC) [18, 19]. Del Rio *et al* identified 14 genes predictive of response to FOLFIRI (5-FU, leucovorin and irinotecan) in a series of 21 patients with mCRC [18], whereas Watanabe *et al* reported a 27-gene prediction model of response to FOLFOX (5-FU, leucovorin and oxaliplatin) in 40 mCRC patients [19]. Of note, none of these small studies attempted to validate the observed results by alternative techniques or in independent patient cohorts. In our study, by contrast, genes identified by whole genome expression analyses to predict response to chemotherapy were further assessed by RT-qPCR in an independent multi-institutional validation cohort. Although direct comparison of genes from predictive signatures reported by Del Rio, Watanabe and our group shows no overlap, common signaling networks were identified to play a relevant role in this context according to the Gene Ontology classification, including cell growth and proliferation, angiogenesis, cell adhesion, immune response and ion/protein transport and binding. A number of reasons may partially explain the lack of consistent results across studies, including heterogeneity in patient characteristics and treatment regimens, study design (prospective versus retrospective), source of predictive tissue, tissue collection procedures, platform used, and statistical and analytical methods. The lack of a control population and of independent validation in a larger cohort of patients is also a major pending issue before this promising tool may be used by oncologists to tailor patient treatment. Our group is currently undergoing a prospective study to validate these findings in paraffin-embedded tissue.

In conclusion, our study identified a 7-gene profile predictive of response to fluoropyrimidine-based chemotherapy in mCRC. This is to our knowledge the first validated predictive profile in advanced colorectal cancer patients. Functional classification of these genes revealed their implication in key pathways of CRC biology, as well as in molecular processes potentially linked to drug sensitivity. As treatment options in CRC continue to expand, the development of predictive signatures shall become invaluable tools to assist clinicians to appropriately select the most effective therapy in each patient and also in providing new clues regarding key molecular pathways involved in drug response. Larger and prospective confirmatory studies are required, however, in order to successfully implement predictive gene-signatures in clinical practice.

## METHODS

### Patients and tumor samples

From 2008 to 2010, patients that met the following inclusion criteria were selected for the present study: 1) histologically confirmed diagnosis of primary CRC; 2) TNM stage IV [32]; 3) treatment with at least one fluoropyrimidine-based chemotherapy regimen for advanced disease; 4) evaluable for response according to RECIST criteria [33]; 5) adequate tissue specimen available for molecular assays (snap-frozen at –80°C with a proportion of tumor cells > 50%). Follow-up was performed in all centers as per ESMO guidelines [34], including a CT scan for response assessment every 8 to 12 weeks in the absence of clinical deterioration or any other clinical suspicion of disease progression. The study protocol was approved by the institutional review boards of participating centers and written consent was provided by all included patients.

Whole genome expression analysis was performed in a training cohort of CRC samples (*N* = 37) collected at the Hospital Marqués de Valdecilla, Santander, Spain. The gene profile was validated by RT-qPCR in an independent multi-institutional cohort that included 53 tumor samples collected at three Spanish hospitals (Hospital Virgen del Rocio (Seville), Hospital Virgen de la Victoria (Malaga) and Hospital de la Merced (Osuna)). Main characteristics of the study population are summarized in supplementary Tables S1 and S2, and are representative of a standard metastatic CRC population. Distribution of clinical and pathological features in the training and validation cohorts did not differ significantly.

### RNA isolation and processing

All tissue samples were preserved at –80°C until RNA extraction and processing. Sample homogenization was achieved using QIAshredder homogenizers and total RNA was extracted using RNeasy Mini kit (both kits from Qiagen Inc; Valencia, CA, USA).

### Microarray gene expression assays

Microarray gene expression assays were performed for each of the 37 samples using Human Whole Genome U133 Plus 2.0 array (Affymetrix Inc, Santa Clara, CA) based on manufacturer's instructions. Following hybridization, arrays were scanned using a GC3000 laser confocal scanner (Affymetrix), and microarray image data were analyzed by GeneChip Operating Software (GCOS 1.4 Affymetrix). Microarray raw data tables have been deposited at the National Center for Biotechnology Information Gene Expression Omnibus (accession number GSE52735).

### Validation of differentially expressed genes by RT-qPCR

Custom-designed TaqMan^®^ Low Density Arrays (TLDA) 7900 HT Micro Fluidic Cards including the 161 genes selected for validation were run and analyzed by the ABI PRISM^®^ 7900HT Sequence Detection System (SDS 2.2, Applied Biosystems) according to manufacturer's protocol.

### Statistical analysis

#### Clinical variables

Descriptive statistics were used to characterize the most relevant clinical parameters. The association of categorical variables was explored by the chi-squared test or Fisher's exact test. To assess distribution of continuous variables among study groups, parametric (*t*-test) or non-parametric tests (Kruskal-Wallis or Mann-Whitney tests) were used when appropriate. Tumor response was evaluated according to the standard RECIST 1.0 criteria [33] to categorize patients as responders ([R]: complete response [CR] + partial response [PR]) or non-responders ([NR]: stable disease [SD] + progression disease [PD]). Progression Free Survival (PFS) was defined as the time elapsed from the date of initiation of first-line chemotherapy to the date of the first documented evidence of disease progression. Overall survival (OS) was calculated from the start of therapy for advanced disease to the date of death from any cause. Survival curves were estimated by the Kaplan-Meier method, and survival differences among groups were assessed by the log-rank test. *p* < 0.05 was considered significant. All analyses were performed using the Statistical Package for the Social Sciences software (SPSS 18.0 for Windows; SPSS Inc, Chicago, IL).

#### Microarrays

Partek Genomics Suite v7.3.1 (Partek Inc.; St. Louis, MO, USA) was used for statistical analysis. Array quality was assessed using the parameters *P call %, Array outlier* and *Normalized Unscaled Standard Error (NUSE)*. Subsequently, data were pre-processed by the RMA (Robust Multichip Average) method. A linear regression model using PCA (principal components analysis) and clustering techniques was done to identify differential gene expression profiles among the two study conditions (responders versus non-responders to first-line chemotherapy for mCRC). As a surrogate marker of chemotherapy response, supervised analysis were also performed to assess differential expression among patients with long versus short progression-free survival (above versus below the median, respectively).

#### qRT-PCR analysis

Cycle threshold (Ct) values were calculated using the SDS software v.2.3 (Applied Biosystems) using automatic baseline settings and a threshold of 0.2. GAPDH was used as endogenous control. Data are presented as target gene expression = 2^−ΔCt^, with ΔCt = (target gene Ct-GAPDH Ct). Gene expression was computed by real-time Statminer^®^ software v.4.2 (Intergromics, Inc), using the Benjamini-Hochberg algorithm [35] with the FDR set at *a* value of 5%. Gene expression had to be detected in at least 50% of samples in each study group in order to be considered for analysis. PCR GEO accession number GSE52513.

## SUPPLEMENTARY TABLES




