# Supplementary material for: Gene expression profile predictive of response to chemotherapy in metastatic colorectal cancer
Source: Oncotarget. 2015 Jan 30;6(8):6151–9. doi: 10.18632/oncotarget.3152 (PMC4467428; doi:10.18632/oncotarget.3152)
Supplement: Supplementary file 1 [file oncotarget-06-6151-s001.pdf]

## SUPPLEMENTARY TABLES

**Supplementary Table S1: Patient characteristics and treatment in training and validation sets**

| | Training set (*n* = 37) | Validation set (*n* = 53) |
|---|---|---|
| | **N (%)** | **N (%)** |
| **Age (years)** | | |
| Median (range) | 61 (47–79) | 65 (42–80) |
| **Sex** | | |
| Female | 16 (43.0) | 18 (34.0) |
| Male | 21 (57.0) | 35 (66.0) |
| **Metastatic disease at diagnosis** | | |
| Yes | 32 (86.5) | 41 (77.4) |
| No | 5 (13.5) | 12 (22.6) |
| **Performance Status** | | |
| 0–1 | 37 (100.0) | 49 (92.5) |
| 2 | 0 (0.0) | 4 (7.5) |
| **Baseline laboratory parameters (Median[range])** | | |
| CEA (ng/mL) | 65 (1–4007) | 15 (1–14948) |
| Haemoglobyn (g/dL) | 12 (2–15) | 12 (8–15) |
| Alkaline phosphatase (UI/L) | 107 (48–620) | 88 (43–1618) |
| Lactate dehydrogenase (UI/L) | 461 (273–2692) | 258 (109–1095) |
| **First-line advanced disease CT regimens** | | |
| Oxaliplatin-based CT | 32 (86.5) | 38 (71.7) |
| Irinotecan-based CT | 0 (0.0) | 12 (22.6) |
| Triplet (Oxaliplatin+Irinotecan+FP) | 5 (13.5) | 0 (0.0) |
| FP alone | 0 (0.0) | 3 (5.7) |
| **Best tumor response to first-line CT** | | |
| Yes (CR + PR) | 23 (62.0) | 27 (50.9) |
| No (SD + PD) | 14 (38.0) | 26 (49.1) |
| **Disease progression after first-line CT** | | |
| Yes | 35 (94.5) | 40 (75.5) |
| No | 2 (5.5) | 13 (24.5) |
| **Surgical resection of metastasis** | | |
| Yes | 9 (24.4) | 13 (24.5) |
| No | 28 (75.6) | 40 (75.5) |
| **Follow-up of alive patients (months)** | | |
| Median (range) | 34.3 (15.9–77.6) | 30.5 (7-77.6) |
| **Patient status at last contact** | | |
| Deceased | 15 (40.5) | 25 (47.2) |
| Alive with tumor | 21 (56.8) | 22 (41.5) |
| Alive without tumor | 1 (2.7) | 6 (11.3) |

CEA: Carcinoembryonic antigen; FP: fluoropyrimidines; CR: complete response; PR: partial response; SD: stable disease; PD: progressive disease.

**Supplementary Table S2: Tumor characteristics in training and validations sets**

| | Training set (*n* = 37) | Validation set (*n* = 53) |
|---|---|---|
| | **N (%)** | **N (%)** |
| **Sample type** | | |
| Surgical specimen | 35 (94.6) | 53 (100.0) |
| Endoscopic biopsy | 1 (2.7) | 0 (0.0) |
| Other | 1 (2.7) | 0 (0.0) |
| **Tumor site** | | |
| Primary tumor location | | |
| Colon | 26 (70.0) | 39 (73.6) |
| Rectum | 11 (30.0) | 14 (26.4) |
| Metastatic locations | | |
| Liver | 30 (81.0) | 29 (54.7) |
| Lung | 11 (30.0) | 12 (22.6) |
| Peritoneum | 6 (16.0) | 11 (20.8) |
| Other | 1 (2.7) | 23 (43.4) |
| **Histologic features** | | |
| Histology | | |
| Adenocarcinoma | 37 (100.0) | 49 (92.5) |
| Mucinous carcinoma | 0 (0.0) | 4 (7.5) |
| Tumor differentiation | | |
| Well differentiated | 14 (37.8) | 24 (45.3) |
| Moderately differentiated | 8 (21.6) | 20 (37.7) |
| Poorly differentiated | 13 (35.1) | 6 (11.3) |
| Unknown | 2 (5.4) | 3 (5.7) |
| Linfovascular invasion | | |
| Yes | 23 (62.2) | 9 (17.0) |
| No | 3 (8.1) | 12 (22.6) |
| Unknown | 11 (29.7) | 32 (60.4) |
| K-ras status | | |
| Wild-type | 17 (45.9) | 26 (49.1) |
| Mutated | 20 (54.1) | 27 (50.9) |
